# Does Insulin Resistance Drive the Association between Hyperglycemia and Cardiovascular Risk?

**DOI:** 10.1371/journal.pone.0039260

**Published:** 2012-06-15

**Authors:** Kristine Færch, Bryan Bergman, Leigh Perreault

**Affiliations:** 1 Steno Diabetes Center, Gentofte, Denmark; 2 University of Colorado Anschutz Medical Campus, Aurora, Colorado, United States of America; Scientific Directorate, Bambino Hospital, Italy

## Abstract

**Background:**

Several studies have shown associations between hyperglycemia and risk of cardiovascular disease (CVD) and mortality, yet glucose-lowering treatment does little to mitigate this risk. We examined whether associations between hyperglycemia and CVD risk were explained by underlying insulin resistance.

**Methods:**

In 60 middle-aged individuals without diabetes we studied the associations of fasting plasma glucose, 2-hour post oral glucose tolerance test plasma glucose, insulin sensitivity as well as body fat percentage with CVD risk. Insulin sensitivity was measured as the glucose infusion rate during a euglycemic hyperinsulinemic clamp, body fat percentage was measured by dual X-ray absorptiometry, and CVD risk was estimated using the Framingham risk score. Associations of fasting plasma glucose, 2-hour plasma glucose, insulin sensitivity and body fat percentage with the Framingham risk score were assessed in linear regression models.

**Results:**

Both fasting and 2-hour plasma glucose levels were associated with higher Framingham risk score (fasting glucose: r^2^ = 0.21; 2-hour glucose: r^2^ = 0.24; *P*<0.001 for both), and insulin sensitivity with lower Framingham risk score (r^2^ = 0.36; *P*<0.001). However, adjustment for insulin sensitivity and 2-hour glucose made the effect of fasting glucose non-significant (*P* = 0.060). Likewise, when adjusting for insulin sensitivity and fasting glucose, the association between 2-hour glucose and Framingham risk score disappeared (*P* = 0.143). In contrast, insulin sensitivity was still associated with Framingham risk score after adjusting for glucose levels (*P*<0.001). Body fat was not associated with Framingham risk score when taking insulin sensitivity into account (*P* = 0.550).

**Conclusion:**

The association between plasma glucose levels and CVD risk is mainly explained by insulin resistance, which raises the question of whether glucose lowering *per se* without changes in the processes that underlie hyperglycemia should be the sole clinical paradigm in the treatment of type 2 diabetes or its prevention.

## Introduction

Type 2 diabetes significantly increases the risk for cardiovascular disease (CVD) and all-cause mortality. People with diabetes without prior myocardial infarction and people with a prior myocardial infarction, but without diabetes, have similar risk of survival [1], and therefore many consider diabetes as a CVD equivalent. Numerous trials have studied effects of intensive glucose-lowering treatment in patients with diabetes, but the results have not been convincing in terms of lowering short-term CVD risk and survival [2]–[4]. These observations raise the question of whether glucose lowering *per se* without changes in the processes that underlie hyperglycemia (*ie.* insulin resistance and/or beta cell failure) should be the sole clinical paradigm in the treatment of type 2 diabetes or its prevention.

Fasting and post-challenge glucose levels do not confer the same risk of CVD disease and mortality. A meta-analysis of several observational studies showed that the association of CVD risk with post-challenge glucose concentration is stronger than that of fasting plasma glucose [5]. These data are supported by the Diabetes epidemiology: collaborative analysis of diagnostic criteria in Europe (DECODE), which showed that 2-hour glucose, but not fasting glucose, predicts CVD mortality in individuals with glucose levels within the normal range [6]. However, it is still unknown which underlying metabolic abnormalities that cause the increased CVD risk in people with elevated 2-hour glucose. Since 2-hour glucose is closely related to peripheral insulin resistance and lack of beta cell compensation [7], insulin resistance is likely to be the link. This suggestion is supported by the European Group for the Study of Insulin Resistance: relationship between insulin sensitivity and cardiovascular disease risk (EGIR-RISC) collaboration, which prospectively evaluates the role of insulin resistance in CVD risk [8]. Also the fact that insulin resistance often clusters with other metabolic and CVD risk factors, such as visceral obesity, hypertension and dyslipidemia [9], makes insulin resistance likely to be responsible for the higher CVD risk in hyperglycemic individuals.

Using the euglycemic hyperinsulinemic clamp in 60 middle-aged individuals without diabetes, we examined whether associations between hyperglycemia and CVD risk were explained by underlying insulin resistance.

## Materials and Methods

### Study population

Data used in this study originate from 3 separate studies that enrolled a total of 60 American men and women without diabetes. Of these, 34 had normal glucose tolerance, 10 had isolated impaired fasting glucose, 6 had isolated impaired glucose tolerance, and 10 had combined impaired fasting glucose and impaired glucose tolerance [10]. BMI ranged from 20.1–41.6 kg/m2 in those with NGT, from 27.8–40.7 kg/m2 in those with i-IFG, from 27.0–37.6 kg/m2 in those with i-IGT and from 24.3–36.8 kg/m2 in those with IFG+IGT. All study procedures took place at the Clinical Translational Research Center at the University of Colorado Anschutz Medical Campus, Aurora, CO, USA between 2004 and 2012.

The studies were performed in accordance with the Helsinki declaration and approved by the Colorado Multiple Institutional review Board. Informed written consent was obtained from all participants prior to the studies.

### Study procedures

#### Oral glucose tolerance test

A standard 75 g oral glucose tolerance test (OGTT) was performed after an overnight fast. Blood samples for measurement of plasma glucose were drawn in the fasting state and 120 min after ingestion of glucose.

#### Euglycemic hyperinsulinemic clamp

On a separate day, peripheral insulin sensitivity was measured by a euglycemic hyperinsulinemic clamp. After an overnight fast, basal blood samples were taken and a 2-hour basal period was initiated. After the basal period, a 2-hour euglycemic hyperinsulinemic clamp at 40 mU/m^2^/min was performed as described previously [11], [12]. Insulin sensitivity was assessed as the mean glucose infusion rate during the last 30 min of the insulin-stimulated steady state period.

#### Cardiovascular risk

CVD risk was calculated using the Framingham risk score, which includes information on gender, age, total and high-density lipoprotein (HDL) cholesterol, diastolic and systolic blood pressure, as well as diabetes and smoking status [13]. Systolic and diastolic blood pressures were measured with the participants in the supine position. Blood samples for measurement of total cholesterol and high-density lipoprotein were taken after an overnight fast. Absolute 10-year risk (%) of developing coronary heart disease (CHD) was calculated, and participants were divided into four categories of risk (very low, low, moderate, and high).

#### Body composition

Body fat percentage and fat-free mass (FFM) was determined by dual-energy X-ray absorptiometry.

### Laboratory analyses

Plasma glucose concentration was analyzed using the hexokinase/G6P-DH technique (Roche Diagnostics, Mannheim, Germany). Serum insulin concentration was analyzed by a radioimmunoassay (Linco Research Inc., St. Louis, MO). Plasma cholesterol and triglyceride concentrations were measured enzymatically using commercially available kits (Beckman Coulter, Inc., Brea, CA).

### Statistical analysis

Linear regression models were used to study the relationships between CVD risk and fasting glucose, 2-hour glucose, insulin sensitivity and body fat percentage. Because absolute 10-year risk of CHD was not linearly related to the measures of glucose metabolism, Framingham risk score was used as outcome in the models. Measures of glucose metabolism and body fat were standardized before analysis to be able to compare the effect size of the different measures on CVD risk. The analyses were performed with and without adjustment for insulin sensitivity, glucose levels and body fat percentage. None of the analyses were adjusted for age and gender, since these were part of the Framingham risk score. All statistical analyses were performed using SAS version 9.1 and *P*<0.05 was considered statistically significant.

## Results

### Clinical characteristics

Clinical characteristics of the study population are shown in Table 1. The population had a wide range of age and obesity, markers of glucose metabolism (fasting glucose, 2-hour glucose and insulin sensitivity) as well as cardiovascular risk factors (Framingham risk score, total cholesterol and HDL cholesterol).

**Table 1:** Clinical characteristics of the 60 study participants.

Men (%)	50
Age (years)	53.0 (41.5;60.5)
BMI (kg/m^2^)	29.5 (26.2;32.7)
Body fat (%)	33.8 (26.3;41.2)
Fasting plasma glucose (mmol/L)	5.2 (4.9;5.7)
2-hour plasma glucose (mmol/L)	5.7 (4.9;7.9)
Glucose infusion rate (mg/min/kg FFM)	4.6 (2.5;7.4)
Systolic blood pressure (mmHg)	132 (121;142)
Diastolic blood pressure (mmHg)	80 (73;85)
Total cholesterol (mmol/L)	4.9 (4.4;5.6)
High-density lipoprotein (mmol/L)	1.2 (0.9;1.4)
Framingham score	7 (2;10)
Absolute 10-year risk of CHD (%)	8 (4;16)
Categories of CHD risk (%)	
Very low risk of CHD (<10%)	51.7
Low risk of CHD (≥10 and <15%)	20.0
Moderate risk of CHD (≥15 and <20%)	10.0
High risk of CHD (≥20%)	18.3

Data are medians (IQR) or percentages. FFM: Fat-free mass.

### Plasma glucose levels and Framingham risk score

**Figure 1:**
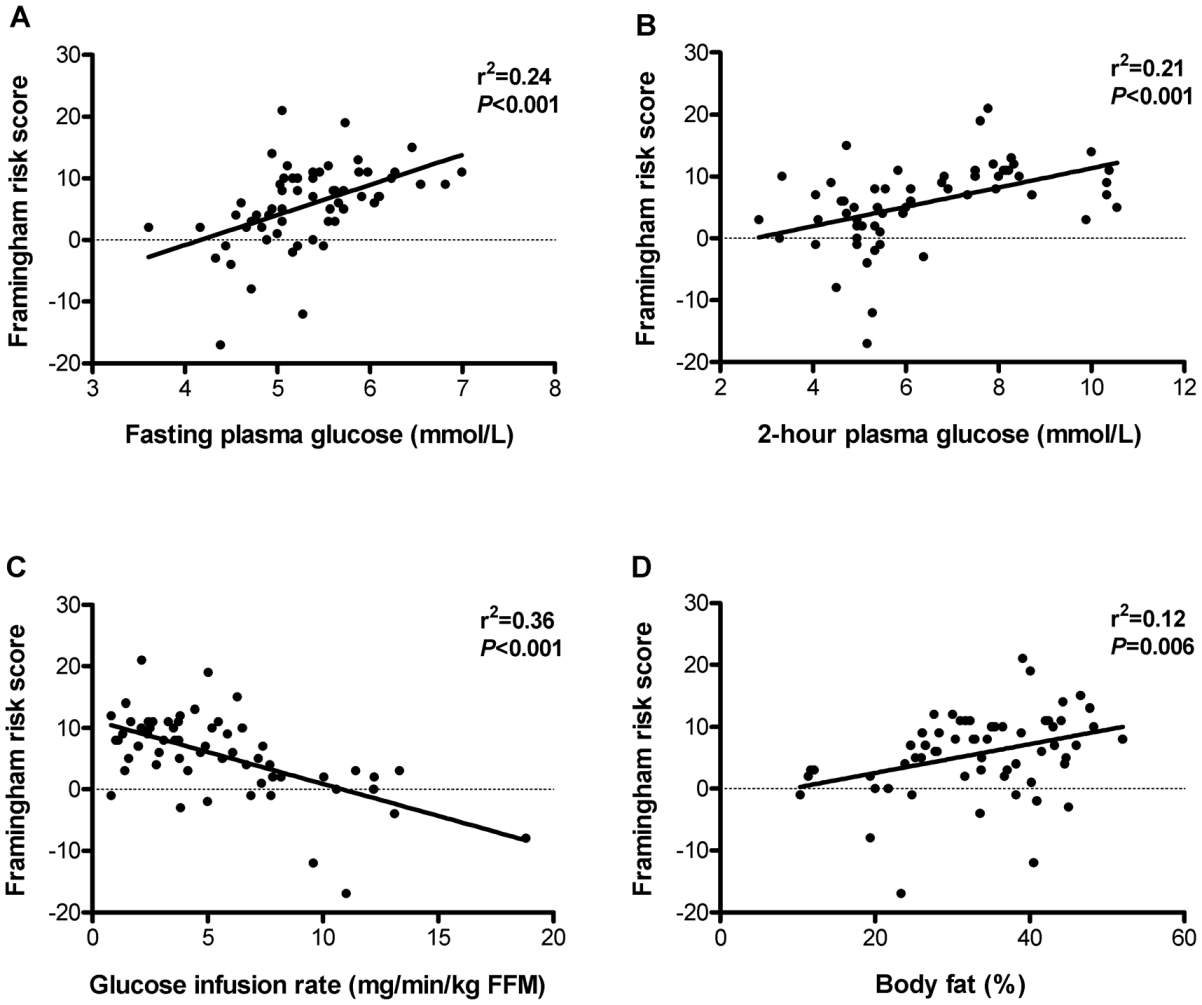
Associations between the Framingham risk score and fasting plasma glucose, 2-hour plasma glucose, insulin sensitivity and body fat percentage. FFM: fat-free mass.

**Figure 2:**
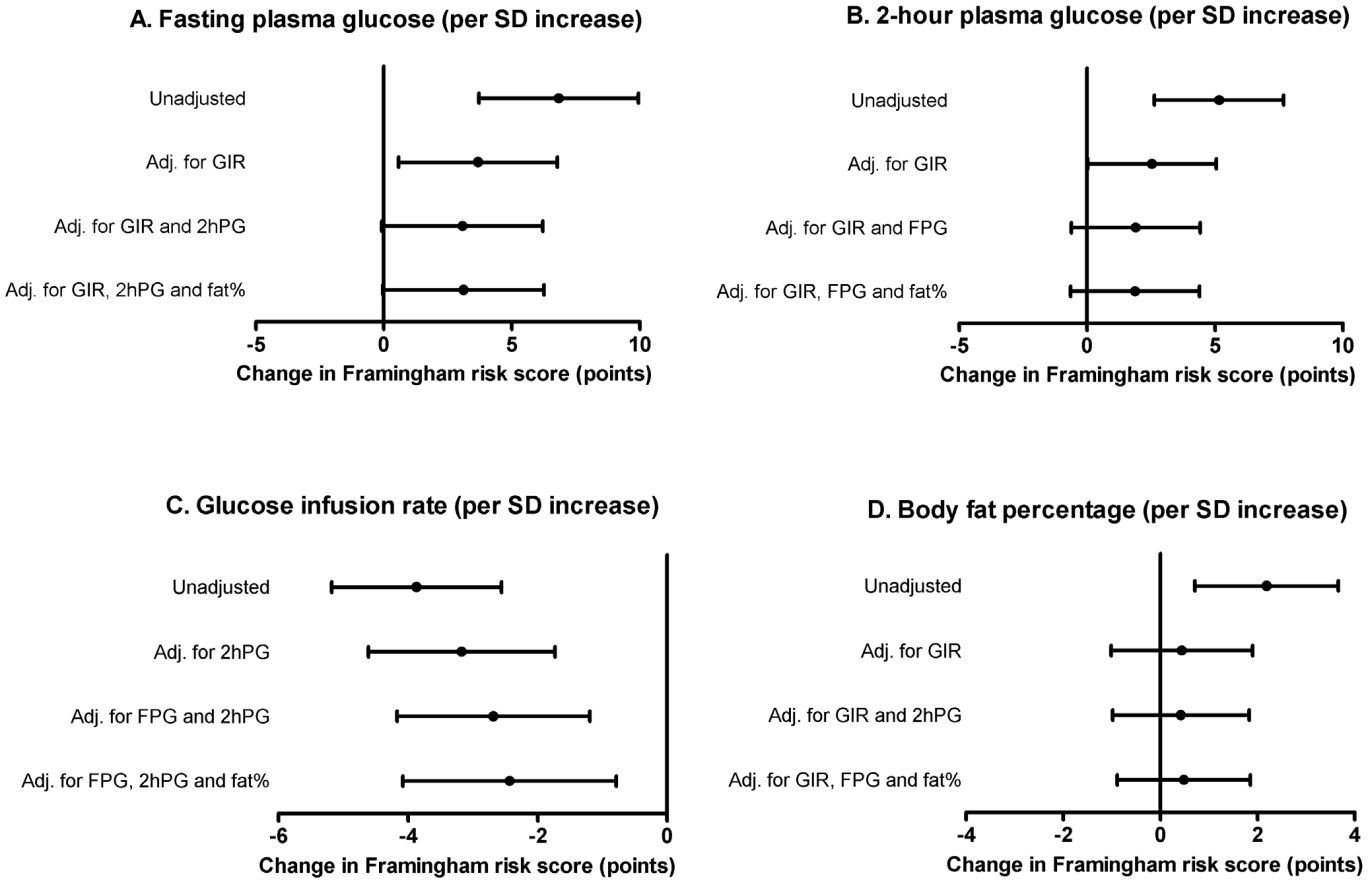
Beta coefficients for relationships between Framingham risk score and fasting plasma glucose, 2-hour plasma glucose, insulin sensitivity and body fat percentage. Beta coefficients reflect the absolute change in Framingham risk score per SD unit increase in the explanatory variables.

Both fasting and 2-hour glucose levels were highly significantly related to the Framingham risk score (*P*<0.001 for both; Figure 1A–B); however, the correlations were not very strong (fasting glucose: r^2^ = 0.24; 2-hour glucose = r^2^ = 0.21). Beta coefficients from the linear regression analyses of Framingham risk score with fasting and 2-hour glucose are shown in Figure 2A–B. The effect sizes correspond to absolute changes in the Framingham risk score per 1 SD increase in the plasma glucose level. In the unadjusted model, 1 SD increase in fasting glucose (∼1.4 mmol/L) was significantly associated with 6.8-point higher Framingham risk score (*P*<0.001; Figure 2A). After adjustment for insulin sensitivity, we only found a 3.7-point higher Framingham risk score per SD increase in fasting glucose (*P* = 0.023), and further adjustment for 2-hour glucose made the association non-significant (*P* = 0.060; Figure 2A). There was a highly significant association between 2-hour glucose and Framingham risk score; 1 SD increase in 2-hour glucose (∼3.3 mmol/L) corresponded to a 5.2-point increase in the Framingham risk score (*P*<0.001; Figure 2B). However, adjustment for insulin sensitivity reduced the strength of the association (*P* = 0.051), and further adjustment for fasting glucose completely abolished the association (*P* = 0.143; Figure 2B).

Adjustment for body fat did not change the associations of fasting and 2-hour glucose with the Framingham risk score (Figure 2A–B).

### Insulin sensitivity and Framingham risk score

Insulin sensitivity was more strongly related to the Framingham risk score than were fasting and 2-hour glucose levels (r^2^ = 0.36, *P*<0.001, Figure 1C). One SD increase in insulin sensitivity (∼3.7 mg/min/kg FFM) was significantly associated with a 3.9-points reduction in the Framingham risk score (Figure 2C). Adjustment for body fat percentage, fasting and 2-hour glucose did only change this association slightly (*P* = 0.005).

### Body fat percentage and Framingham risk score

Body fat was significantly related to the Framingham risk score in the unadjusted model, but the correlation was very weak (r^2^ = 0.12; *P* = 0.006; Figure 1D); 1 SD increase in body fat percentage (∼9.4%-points) corresponded to 2.2-points higher Framingham risk score (Figure 2D). No association between body fat and Framingham risk score was observed after adjustment for insulin sensitivity (*P* = 0.550) or additionally plasma glucose levels (*P* = 0.492).

## Discussion

Several studies have demonstrated relationships of plasma glucose levels with CVD and mortality [6], [14]–[17], yet glucose-lowering treatment does little to mitigate this risk. In this study we hypothesized that underlying insulin resistance drives the associations between glycemia and CVD risk. By using gold standard measures of insulin sensitivity, we demonstrated that insulin resistance is largely responsible for the relationship between plasma glucose levels and CVD risk. We also showed that insulin resistance independent of plasma glucose levels and obesity is strongly associated with CVD risk as estimated by the Framingham risk score.

Over the past decades, a considerable number of intervention studies have focused on the role of glucose-lowering strategies on CVD morbidity and mortality in patients with type 2 diabetes [2]–[4]. The results from these trials have only shown limited beneficial short-term effects of intensive glucose-lowering treatment. Two large trials did not show beneficial effects of 5-years intensive glucose-lowering treatment on major cardiovascular outcomes [2], [3]. Moreover, the Action to Control Cardiovascular Risk in Diabetes (ACCORD) trial demonstrated that intensive lowering of glycemia increased mortality and did not reduce major CVD events [4]. A 10-year follow-up of the UK Prospective Diabetes Study (UKPDS) did show beneficial effects of treatment with sulfonylurea and insulin on CVD and deaths from any cause. However, of interest, patients only treated with the insulin-sensitizing agent metformin seemed to have at least at as favorable effects as those treated with sulfonylurea and insulin [18].

Our results indicate that lowering hyperglycemia without changing the underlying processes that cause hyperglycemia may not be the optimal way of reducing CVD risk. Thus, treatment modalities for improvement of insulin resistance may be more efficient both in terms of reducing CVD risk and improving glycemic control. Indeed, there are indications that treatment strategies focused on improving insulin sensitivity have beneficial effects on CVD risk factors and events. The Actos Now for Prevention of Diabetes (ACT NOW) study showed beneficial effects of the insulin-sensitizing agent pioglitazone on carotid intima-media thickness in individuals with impaired glucose tolerance [19]. Also the PROspective pioglitAzone Clinical Trial In macro-Vascular Events (PROactive) [20] and the Pioglitazone Effect on Regression of Intravascular Sonographic Coronary Obstruction Prospective Evaluation (PERISCOPE) [21] showed that treatment with pioglitazone decreased major CVD events and progression of atherosclerosis in type 2 diabetes patients with pre-existing CVD. In addition to these pharmacological trials, beneficial effects of intensive lifestyle-modification on CVD risk factors have been observed both in individuals with diabetes and pre-diabetes [22], [23].

To our knowledge, this is the first study to address whether gold-standard-measured insulin sensitivity drives the association between glycemia and CVD risk. In the San Antonio Heart Study, insulin resistance as measured by the homeostasis model assessment (HOMA-IR) was related to CVD risk factors, whereas insulin secretion was not [24]. Interestingly, they also found that insulin-sensitive individuals who developed diabetes during 7-years of follow-up had a CVD risk similar to people who did not develop diabetes [24]. This indicates that hyperglycemia *per se* was not related to CVD risk. This finding is in accordance with a recent study showing that physical activity reduces the risk of CVD death and all-cause mortality across a wide spectrum of glycemic control including people with diabetes [25]. The authors found that physically active individuals with hemoglobin A_1c_ (HbA_1c_) levels ≥7% did not have increased risk of CHD or mortality. The long-term effects of insulin resistance on CVD risk is currently being investigated in the EGIR-RISC study [8].

We found that insulin sensitivity as measured by the clamp technique explained more of the relationship between 2-hour glucose and Framingham risk score than between fasting glucose and Framingham risk score. The reason for this may be related to the underlying physiology of fasting and post-OGTT hyperglycemia. Elevated 2-hour glucose is mainly related to peripheral insulin resistance and beta cell decompensation, whereas elevated fasting glucose is predominantly caused by hepatic insulin resistance and reduced first-phase insulin secretion [7]. Insulin sensitivity estimated by the clamp technique predominantly reflects peripheral insulin sensitivity [26], which may explain why it does not fully account for the association between fasting glucose and CVD risk. Future studies may address whether adjustment for hepatic insulin sensitivity further deteriorates the association between fasting glucose and risk of CVD.

There are several limitations related to the current study. First, because of the cross-sectional nature of this study we cannot determine whether insulin sensitivity in fact is related to future CVD outcomes. Second, because we did not measure beta cell function in the entire study population, we cannot exclude disturbances of insulin secretion being responsible for part of the observed findings. Third, the use of the Framingham risk score has several limitations. In general, risk engines are thought to underestimate risk in women compared with men [27]. Moreover, the Framingham risk score does not predict CHD risk equally in all ethnic groups [28]. It has also been suggested that the diabetes-specific UKPDS risk engine has several advantages over the Framingham risk score, because it includes a measure of glycemia (HbA_1c_) as well as diabetes duration in addition to the traditional risk factors [29]. However, a systematic review demonstrated that there is not evidence to suggest that diabetes-specific risk assessment tools are superior to risk engines developed in the general population [30]. Moreover, since we estimated CVD risk in people without diabetes, the Framingham risk score seems to be a better choice than the UKPDS risk engine. Of note, both the UKPDS and the Framingham risk score overestimate absolute CVD risk as compared to observed CVD outcomes, but the ability of the equations to rank individuals according to CVD risk seems to be modest [31], which is of importance in this particular study.

In summary, we demonstrated that insulin resistance is responsible for a large part of the associations between plasma glucose levels and CVD risk. If these data are confirmed in other larger prospective studies, markers of insulin resistance (beyond glucose) should be explored for their clinical utility in high-risk individuals before the onset of diabetes.
